# Phytohormones and Shoot Branching: A Mini-Review of Molecular Regulatory Networks and Mechanisms

**DOI:** 10.3390/plants15142197

**Published:** 2026-07-17

**Authors:** Xingxing Zhang, Miao Chai, Yijia Wu, Jialei Tang, Cong Yin, Lu Zhang, Shuai Gao

**Affiliations:** 1 The Key Laboratory of Quality and Safety Control for Subtropical Fruit and Vegetable, Ministry of Agriculture and Rural Affairs, College of Horticulture, Zhejiang A & F University, Hangzhou 311300, China; 20220044@zafu.edu.cn (X.Z.); 2023114012004@stu.zafu.edu.cn (M.C.); 986621@stu.zafu.edu.cn (Y.W.); 865533@stu.zafu.edu.cn (J.T.); 2 Haining High-Tech Research Institute, Haining, Jiaxing 314400, China; sallyyc@hotmail.com

**Keywords:** lateral branch, axillary meristems, phytohormones, regulatory mechanism

## Abstract

As a crucial component of plant architecture, shoot branching significantly enhances photosynthetic efficiency and optimizes the source-sink allocation of photosynthetic products, which directly impacts crop productivity. The formation of lateral branches is co-regulated by various internal and external factors, including genetic factors, phytohormones, metabolic, and environmental factors. In this review, we provide a mechanistic overview of the key regulatory events involved in axillary meristem (AM) formation and lateral branch development, emphasizing the significant regulatory roles of phytohormones (such as auxins, cytokinins, strigolactones, brassinosteroids, gibberellins, abscisic acid, and other phytohormones) in this process. Furthermore, we highlight the transcription factors SHOOT MERISTEM-LESS (STM) and BRANCHED1 (BRC1) as central hubs that integrate multiple hormonal signals to control AM initiation and lateral branch development, respectively. By elucidating the regulatory mechanisms of these key nodes, we propose future research directions that can provide a foundation for shaping ideal crop plant architecture through genetic engineering and breeding strategies.

## 1. Introduction

Different plants exhibit a diverse array of visual appearances and structural features in nature. These distinct characteristics enable successful identification and differentiation among various species. During the long-term evolutionary process, the unique traits of plants provide a robust foundation for their adaptation to specific habitats and facilitate the alternation of generations [1]. Plant architecture, which encompasses aspects such as height, stem branching, inflorescence branching, and leaf angle, is one of the most intuitive and significant structural components of plants [2,3]. Plant architecture is an important agronomic trait that influences the growth, yield, and stress resistance of crops [4,5,6,7,8,9]. For field crops, an ideal plant architecture enhances efficient light capture, thereby improving photosynthetic efficiency and creating favorable conditions for the accumulation of photosynthetic products. Conversely, it can also influence the distribution of nutrients among different parts of the plant, impacting the growth and development of various organs, which ultimately results in increased plant biomass and crop yield [10,11]. For ornamental plants, specific plant architectures are often associated with higher economic value. Therefore, genetic improvement of plant architecture in cultivated crops and ornamental plants is crucial for enhancing agricultural production efficiency. Lateral branches are a crucial component of plant architecture and represent a significant agronomic trait of concern in plant production. For example, during the protected cultivation of tomatoes, excessive lateral branching can lead to mutual shading, thereby reducing overall photosynthetic efficiency. Additionally, it hinders ventilation within the facility, promoting disease and disrupting the distribution of photosynthetic products, which ultimately reduces yield and quality [12]. In the cultivation of ornamental plants, branching holds esthetic value and can enhance the ornamental quality of the flowers [13]. Furthermore, in monocotyledonous crops such as rice and wheat, an increase in effective tillers (lateral branches formed at the base of the stem) can enhance the number of panicles, contributing to increased yield [14,15]. Therefore, the effects of branching differences vary among different crops during plant production.

The development of lateral branches progresses through four distinct stages: AM formation, bud formation, activation, and sustained outgrowth, ultimately resulting in the emergence of lateral branches [16]. During the AM formation stage, SHOOT MERISTEM-LESS (STM) serves as the primary regulator, which is predominantly controlled by the LATERAL SUPPRESSOR (LAS)-REVOLUTA (REV)-DORNROSCHEN (DRN) and REGULATOR OF AXILLARY MERISTEMS (RAX)-CUP-SHAPED COTYLEDON (CUC) pathways [17,18]. STM facilitates AM formation by enhancing the synthesis of cytokinins (CKs). WUSCHEL (WUS) and CLAVATA 3 (CLV3) establish a negative feedback loop that collectively regulates the activity and stability of AM. The upregulation of *STM* expression promotes CK synthesis, which subsequently activates *WUS* expression through the CK signaling pathway, thereby facilitating bud formation [19,20]. Concurrently, auxins produced in the shoot apex inhibit bud formation by suppressing CK levels indirectly. Once buds are formed and conditions become favorable, they transition into the activation stage. BRANCHED 1 (BRC1), a key inhibitor of lateral bud growth, integrates various hormonal signals and restricts lateral bud growth either by inhibiting auxin efflux from the bud or by promoting abscisic acid (ABA) synthesis within the bud. CKs and brassinosteroids (BRs) downregulate *BRC1* expression and promote lateral bud growth, whereas auxins enhance *BRC1* expression through CKs and strigolactones (SLs) indirectly, thus inhibiting lateral bud growth [21,22]. During the sustained bud growth stage, increased auxin synthesis and polar auxin transport (PAT) contribute to the direct promotion of lateral bud growth, while also enhancing gibberellin (GAs) synthesis [23]. Furthermore, ethylene promotes lateral bud growth by inhibiting ABA metabolism, whereas jasmonic acid (JA) suppresses bud growth by enhancing BRC1 expression [24].

To date, the analysis of multiple mutants has significantly advanced our understanding of shoot branching, and the regulatory mechanisms are becoming increasingly clear. A comprehensive understanding of the regulatory mechanisms governing shoot branching is essential for improving plant architecture and enhancing crop production efficiency. To this end, we conducted a search of the PubMed and Web of Science databases, collecting and summarizing the recent progress in understanding the effects of phytohormones on shoot branching, and offering insights into future research directions.

## 2. The Processes of Lateral Branches Development

**Figure 1:**
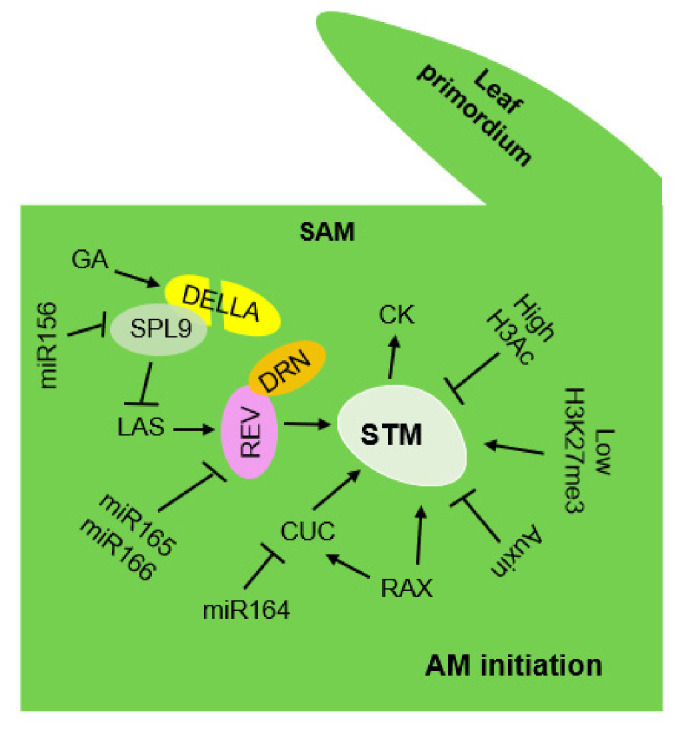
The molecular interaction network of AM initiation. As the core regulator of AM initiation, *STM* expression is co-regulated by the LAS-REV-DRN pathway and the RAX-CUC pathway. Concurrently, miR156, miR164, and miR165/166 are integrated into these two pathways through their respective target genes *SQUAMOSA PROMOTER-BINDING-PROTEIN-LIKE* (*SPL9*), *REV*, and *CUC*, thereby regulating *STM* expression. The DELLA-SPL9 interaction module further mediates the regulation of LAS by GA signaling. Furthermore, auxin and elevated levels of H3AC inhibit *STM* expression, whereas low levels of H3K27me3 promote it. STM facilitates the initiation of AM by enhancing cytokinin synthesis. Lines with arrows and blunt ends denote positive and negative regulation, respectively.

The development of lateral branches in plants is an extremely complex process. It involves three main stages, including the formation of the AM (Figure 1), and the formation, activation, and outgrowth of axillary buds. Axillary branches originate from the AM.

*STM* encodes a KNOX transcription factor and is a key factor regulating AM initiation [25,26,27]. In the AM, the loss of STM prevents axillary bud formation. Only a small population of cells in the leaf axil can maintain *STM* expression to retain meristematic identity, which is essential for AM initiation. *STM* expression is regulated by a complex network, with LAS, RAX, and CUC forming the core of this regulatory network. Research shows that the HD-ZIP III transcription factor REV, acting downstream of *LAS* promotes the expression of *STM* through its interaction with the AP2/ERF protein DRN/DRN-like [28,29]. Other studies have also shown that *CUC* and *RAX* genes regulate the expression of *STM* via LAS [30]. During axillary bud formation, CLV3 and WUS regulate the morphological development of the shoot apical meristem through a negative feedback loop. Additionally, epigenetic regulation plays an important role in controlling gene expression in plants [31]. Recent studies have revealed that the STM gene exhibits high levels of H3Ac and low levels of H3K27me3 in AM tissues [32]. Chromatin assembly factors FAS1 and FAS2 restrict the activity of the *WUS* gene [33]. In addition, miR165/166 plays an important role in the initiation of AM. The *Arabidopsis argonaute 10* (*ago10*) mutant exhibits defects in AM initiation, and in the early stage of AM initiation, AGO10 binds miR165/166 and causes its degradation, while the miR165/166 target gene *REV* is activated, thereby achieving precise temporal and spatial regulation of *REV* expression during AM initiation [34]. After the axillary bud is formed, it continues to develop into an axillary bud. Under unfavorable conditions, the axillary bud temporarily enters a dormant state, and resumes growth when the nutritional status and hormonal conditions become suitable. The transcription factor BRC1 is a core repressor of axillary bud growth, and its expression regulation is jointly controlled by hormonal signals, metabolites, and environmental signals. In addition, due to the effect of apical dominance, the growth and formation of axillary buds are inhibited, which is a key factor influencing axillary bud dormancy or activation [35]. In Arabidopsis, mutations in YUCCA lead to reduced IAA synthesis capacity, resulting in the loss of apical dominance and the production of dense lateral branches [36]. Achieving precise regulation of apical dominance is an important step in improving crop plant architecture.

## 3. The Hormonal Regulation in Shoot Branching

**Figure 2:**
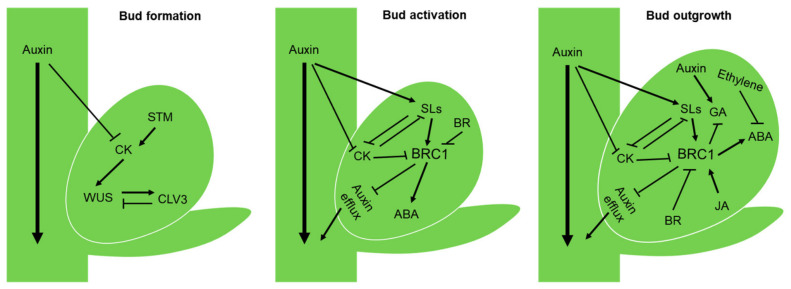
Regulatory model of phytohormones in shoot branching. Shoot branching can be divided into three stages: bud formation, activation, and continuous outgrowth. WUS and CLV3 form a negative feedback loop that collectively determines meristem activity and stability. During the bud formation stage, the upregulation of *STM* expression promotes CK synthesis, which in turn activates *WUS* expression and facilitates bud formation. Meanwhile, auxin produced at the shoot apex inhibits bud formation by suppressing CK biosynthesis. In the bud activation stage, BRC1 functions by either inhibiting auxin efflux from the bud or promoting ABA synthesis in the bud. Auxin produced by the shoot apex promotes *BRC1* expression by inhibiting CK and promoting SLs, thereby inhibiting auxin efflux in buds. SLs upregulate *BRC1* expression, while CK and BRs inhibit *BRC1* expression. In the outgrowth stage, enhanced auxin synthesis and polar transport directly promote the continuous growth of lateral buds, while also further stimulating lateral bud growth through the promotion of GAs. Ethylene inhibits ABA levels, while JA promotes *BRC1* expression. Lines with arrows and blunt ends denote promotion and repression, respectively.

Phytohormones play essential roles in plant growth and development. All three stages of bud development are precisely and intricately regulated, with hormones playing an indispensable role in this process (Figure 2). Here, we outline the functions of phytohormones in regulating shoot branching and the molecular mechanisms underlying their actions, aiming to deepen the understanding of hormone-mediated regulation of shoot branching.

### 3.1. Auxins

Auxins function to regulate plant cell division and differentiation and play an important role in the formation of AM. Local biosynthesis and PAT establish auxin maxima and minima, which guide cell fate decisions critical for meristem formation, lateral organ initiation, and morphogenesis. Studies indicate that auxin does not directly act on axillary buds; rather, it suppresses bud activity by regulating the downstream auxin signaling pathway repressor INDOLE-3-ACETIC ACID 15 (IAA15) [37]. Additionally, loss of function of the auxin efflux gene *PIN4* results in increased auxin content in the leaf axil, thereby inhibiting lateral branch formation [37]. Studies indicate that in apical dominance, PAT in the main stem is mediated by PINFORMED (PIN) auxin efflux facilitators located in xylem-associated cells [38], which exert an inhibitory effect on lateral bud growth [39,40,41]. Due to insufficient auxin transport from the stem to the bud, auxin cannot directly regulate the expression of *BRC1* [42]. However, some studies have suggested that auxin can indirectly promote the expression of *BRC1* in lateral buds [43]. Recent research has also demonstrated that temperature can enhance the expression of genes related to auxin synthesis and signal transduction by activating the TEOSINTE BRANCHED1/CYCLOIDEA/PROLIFERATING CELL FACTOR 15 (TCP15)—HISTONE DEACETYLASE 4 (HDA4) module, subsequently inhibiting the germination of tomato lateral branches [44]. This suggests that epigenetic modifications play a role integrating auxin-mediated environmental signals. Furthermore, the miR156-targeted *SBP15* transcription factor plays a role in the inhibition of auxin-mediated shoot branching by regulating hormone dynamics and interacting with factors such as GOBLET and BRC1 [45]. Poplar PagKNAT2/6b responds to apical stress treatment by inhibiting the expression of the auxin synthesis gene *YUC6a*, thereby reducing IAA content in plants, which directly leads to decreased expression of strigolactone synthesis genes MORE AXILLARY GROWTH 3 (*MAX3*) and MORE AXILLARY GROWTH 4 (*MAX4*), reducing SL content and promoting branching [46]. In cucumber, during domestication, the increased level of *CsBRC1* directly inhibits the activity of PIN-FORMED 3 (PIN3) in lateral branches, leading to excessive accumulation of auxin in lateral branches, thereby suppressing their growth and development of lateral branches [47].

### 3.2. Cytokinins

Cytokinins play a critical role in promoting the growth and development of plants. In lateral branch development, cytokinins act as signaling molecules in the regulation of AM initiation, meristem activity, and apical dominance-mediated lateral bud growth. Recent studies have shown that the perception and signal transduction of CKs are essential for the formation of AM [48]. In addition, CKs play a positive role in the activation of dormant buds, and this effect is directly related to the CK content of the buds [49]. CK and STM can form a positive feedback loop [2,48,50]. Ectopic expression of the cytokinin biosynthesis gene *IPT8* in leaf axils partially rescued the defective AM initiation phenotype in the *rax1 rax2 rax3* triple mutant, indicating that cytokinin plays a positive role in AM [48,50]. Furthermore, in the regulation of meristem activity, CKs can activate type-B response regulators through their receptor ARABIDOPSIS HISTIDINE KINASE (AHK), which in turn directly activates the expression of *WUS*, thereby influencing meristem activity [48,51,52]. In highly defined meristems, CKs can regulate the expression of *WUS*, and the binding of WUS and CKs is associated with increased histone acetylation and methylation marks, indicating that the CK pathway regulates plant branching in a tissue-specific manner and is linked to epigenetics [52,53]. In addition, CKs are key hormones that alleviate apical dominance and promote the activation of lateral buds. In Arabidopsis, meristem functional mutants exhibit a phenotype of higher CK levels, enhanced shoot development, an increased number of AMs, and reduced *BRC1* expression levels. Moreover, the expression of CK synthesis genes in tomato lateral buds is upregulated after topping, resulting in increased CK content [21]. And, overexpression of CK biosynthesis genes in tomato significantly reduces apical dominance [54]. a result that was also confirmed by exogenous CK addition experiments [55]. The KNOX-type homeobox protein RICE LATERAL BRANCHING (RLB) can recruit the Polycomb Repressive Complex 2 (PRC2) member OsEMF2b to the promoter of the cytokinin oxidase gene CYTOKININ OXIDASES 4 (*OsCKX4*), thereby inhibiting the expression of *OsCKX4* and promoting lateral branching in rice [56]. In summary, CKs regulate the development of lateral branches in plants by controlling the expression of genes involved in AM initiation, meristem activity, and apical dominance.

### 3.3. Strigolactones

Strigolactones are a class of sesquiterpenoid plant hormones that are mainly synthesized in stems and roots [57,58]. They were discovered for their ability to promote the germination of seeds of parasitic plants such as Striga and Orobanche [59,60]. In recent years, researchers have identified multiple genes involved in SL biosynthesis and signal transduction from highly branched mutants, suggesting that SLs play an important regulatory role in plant lateral branch development [61]. In general, reducing or eliminating the production or signal transduction of SL leads to the transition of axillary buds from a dormant state to an active growth state, ultimately promoting the growth of lateral branches [61]. For example, mutations in carotenoid cleavage dioxygenases 7 and 8 (CCD7 and CCD8), two enzymes involved in SL biosynthesis, result in reduced branching in tomato and exhibit similar effects in different species including Arabidopsis, apple, pea, rice, kiwifruit, and orchardgrass [62,63,64,65,66,67,68]. In tobacco, Methyl-CpG BINDING DOMAIN PROTEIN 1 (*NtMBD1*) encodes a putative cytochrome P450 gene, and its mutation leads to reduced plant height and increased lateral branch elongation. Further analysis showed that MBD1 indeed participates in the regulation of plant branching through the strigolactone signaling pathway [69].

The effect of SLs on branching is mediated through the SL signaling pathway. This pathway ubiquitinates and degrades downstream repressors SUPPRESSOR OF MORE AXILLARY GROWTH 2 LIKE (SMXLs)/PhD53A/DWARF 53 (D53) via the SL receptor (AtD14/DAD2/RMS3/D14) and the F-box complex (MAX2/PhMAX2A/RMS4/D3), thereby activating downstream transcription factors such as BRC1, IDEAL PLANT ARCHITECTURE1 (IPA1) [57,70]. For instance, D53/D53-like proteins regulate the expression of downstream genes such as BRC1 by interacting with SPL transcription factors or by directly acting as transcription factors [71,72]. Moreover, SLs activate the transcription of *MdHB53* and *MdTCP18* through the MdSMXL7-MdSPL6 interaction module, thereby increasing ABA levels to inhibit axillary bud germination in apple [73]. In addition, PLEIOTROPIC DRUG RESISTANCE 1 (PDR1) can transport SLs over long distances, thereby regulating lateral branch development in Petunia hybrida; however, the specific mechanism remains to be elucidated [74]. Therefore, SLs are crucial for the optimization of plant architecture. The regulation of shoot branching by SLs is mainly manifested in two aspects: one is to regulate the number of lateral branches by controlling the initiation of lateral buds [such as MONOCULM 1 (MOC1), LAX PANICLE 1/2 (LAX1/2), SIRTUIN 1 (SRT1) ect.]; the other is to affect plant tillering by regulating the growth of axillary buds (such as D3, D14, D53, SMXLs ect.) [75,76,77,78]. In summary, the regulatory mechanism of SLs on shoot branching is relatively well understood.

### 3.4. Brassinosteroids

Brassinosteroids, as important plant growth-regulating hormones, play a key role in plant morphogenesis, seed germination, fruit ripening, and stress resistance [79,80,81,82,83,84]. To date, the role of BRs in regulating shoot branching has been clarified, and BRs exert a promoting effect on this process. In Arabidopsis, the gain-of-function mutant bes1-D exhibits a phenotype of increased rosette leaf branching, whereas defects in BR biosynthesis and signaling result in a reduction in tiller number [85,86]. Furthermore, a study in tomato found that BRs are key signals for releasing apical dominance, promoting lateral bud activation and lateral branching by integrating hormonal and sugar signals [22]. The transcription factor *SQUAMOSA PROMOTER-BINDING-PROTEIN-LIKE 13* (*SlSPL13*), encoded by a target gene of miR156a, regulates tomato lateral branching by directly binding to the promoters of *BRC1b* and *DWF*, promoting *BRC1b* expression while inhibiting *DWARF* (*DWF*) expression [87]. Furthermore, heterologous overexpression of pepper BRASSINAZOLE-RESISTANT 1.2 (CaBZR1.2) increases the number of lateral branches in tomato [88]. In chrysanthemum, BASIC LEUCINE ZIPPER 19 (CmbZIP19) inhibits lateral bud elongation by downregulating the expression of the BR biosynthesis gene *CmDWF1*, whereas the lncRNA1-miR6288b-3p-PpTCP4-PpD2 module regulates branch number by modulating brassinosteroid biosynthesis in peach [89]. In addition, recent studies have also found that BRI1-EMS-SUPPRESSOR 1 (BES1) or BRASSINAZOLE-RESISTANT 1 (BZR1) interact with D53-like proteins to form a complex, inhibiting the expression of *BRC1* in *Arabidopsis* or FINE CULM 1 (*FC1*) in rice, thereby promoting shoot branching [72,90]. This indicates that BRs regulate shoot branching primarily in relation to SLs, and these two phytohormones play opposite roles in shoot branching.

### 3.5. Gibberellin Acid

Gibberellin acid is an important phytohormone regulating plant growth and development in plants [91]. GA exhibits varying effects on the regulation of lateral branch development across different species. In Arabidopsis, GA exerts an inhibitory effect by promoting the degradation of DELLA proteins, which function as repressors, through the activation of its own signaling pathway. DELLA proteins typically inhibit the expression of *LAS*, a key gene for lateral bud formation, by interacting with the transcription factor SPL9. Following GA treatment, the degradation of DELLA proteins releases SPL9, which subsequently represses *LAS* transcription and hinders lateral bud initiation [92]. Additionally, the GA signaling pathway can enhance the activity of the APC/C^TE^ complex by antagonizing ABA signaling, leading to the degradation of the MOC1 protein and consequently inhibiting tillering in rice [93]. Conversely, in various woody plants such as poplar and peach, GA serves as an activator of lateral bud growth [94]. Research indicates that the branching-promoting effect of CKs necessitates the involvement of GA; treatment with the GA biosynthesis inhibitor paclobutrazol (PAC) significantly diminishes the branching-promoting effect of CKs [94]. In hybrid poplar, the dynamic balance between the two bioactive forms, GA_3_ and GA_4_, regulates the dormancy and germination of lateral buds: dormant buds exhibit elevated levels of GA_3_ and GA_6_, which induce *GIBBERELLIN 2-OXIDASE* (*GA2ox*) gene expression to convert active GA_4_ into inactive forms, thereby maintaining dormancy; following the release of apical dominance, GA_4_ biosynthesis rapidly increases, promoting lateral bud elongation [95]. In tobacco, NtMAB1-S1 significantly increases the content of trehalose-6-phosphate (Tre6P) and active GAs by inhibiting the *TREHALOSE-6-PHOSPHATE PHOSPHATASE F* (*NtTPPF*) and *NtGA2ox8* expression, thereby activating dormant axillary buds and promoting sustained growth [96]. In rice, GA-deficient mutants exhibit a high-tiller phenotype, while mutations in the negative regulator of GA signaling, DELLA, lead to a reduction in tiller number, highlighting the complexity of GA and its signaling in tillering regulation [97,98]. In addition, high concentrations of GA can also promote the degradation of MOC1 in the AM by activating APC/C^TE^, thereby inhibiting rice tillering [93]. In summary, the regulation of branching by GA is very complex and exhibits clear species specificity.

### 3.6. Abscisic Acid

Abscisic acid, known as a stress hormone due to its massive production after plants are subjected to stress, not only regulates plant stress resistance but also plays an irreplaceable role in plant growth and development [99,100]. ABA mainly plays an inhibitory role in shoot branching. For example, studies in rice, Arabidopsis, soybean, cotton, pear, and wheat have shown that ABA inhibits tillering/branching in these plants [101,102,103,104,105]. Further analysis in *Arabidopsis* revealed that BRC1 can bind and activate the transcription factors *HB21*, *HB40*, and *HB53*, promoting the expression of the ABA biosynthesis gene *NCED3*, increasing ABA content, and thereby enhancing the dormancy of axillary buds [106]. This study directly links BRC1 to ABA, providing strong evidence that ABA is a general inhibitor of bud growth. In cucumber, the MADS-box transcription factor CsAGL16 reduces ABA levels in axillary buds and promotes axillary bud elongation by directly binding to and enhancing the expression of the ABA 8′-hydroxylase gene *CsCYP707A4* [107,108]. Subsequently, the same research group also found that the CsphyB-CsPIF4-CsBRC1 module regulates branching by integrating light signaling and ABA biosynthesis [109]. In addition to ABA metabolism, changes in ABA signaling also affect bud dormancy and lateral shoot growth. ABA inhibits the activity of the APC/C^TE^ complex by SnRK2 phosphorylation, thereby enhancing the stability of MOC1, and ultimately promoting the formation of lateral branches in rice [93]. In wheat, Tiller Number1 (TN1), on one hand, inhibits the expression of key genes in ABA synthesis and promotes ABA accumulation; on the other hand, TN1 interacts with TaPYL (ABA receptor), thereby inhibiting the interaction between TaPYL and its downstream key ABA signal transduction protein TaPP2C, interfering with ABA signaling, and ultimately promoting the elongation of wheat tiller buds [104]. In summary, ABA biosynthesis and signaling pathways act together downstream of BRC1 to jointly regulate plant bud dormancy and lateral branch growth.

### 3.7. Other Hormones

To date, research on the regulation of plant branching by the plant hormones ethylene and JAs has been very limited, and studies concerning salicylic acid (SA) are virtually nonexistent. Therefore, here we primarily summarize shoot branching mediated by ethylene and JAs. For ethylene, the core key transcription factor ETHYLENE INSENSITIVE 3 (CsEIN3) in the ethylene pathway promotes branch development by activating *CsCYP707A4*, while inhibiting tendril growth by suppressing *TENDRIL-LESS 1* (*CsTL*) expression in cucumber [110]. In rice, *GRAIN NUMBER PER PANICLE 3* (*GNP3*) encodes a MAPK kinase kinase (OsMKKK22) that promotes an increase in panicle branch number and grain number per panicle by phosphorylating S-adenosylmethionine synthetase 1 (SAMS1), facilitating its degradation, and thereby inhibiting ethylene biosynthesis [111]. For JA, drought stress activates *MYELOCYTOMATOSIS PROTEINS 2* (*CmMYC2*) by inducing JA accumulation, and CmMYC2 directly binds to the *CmBRC1b* promoter and activates its expression to inhibit axillary bud development in chrysanthemum [24]. JAs activate the zinc finger transcription factor ZINC FINGER OF ARABIDOPSIS THALIANA 11 (GhZAT11). Subsequently, GhZAT11, on one hand, directly activates the expression of *SUCROSE TRANSPORTER 4* (*GhSUT4*), accelerating the efficient transport of sugars from storage organs to dormant buds; on the other hand, it directly activates the expression of *CYCLIN D2;1* (*GhCYCD2;1*), promoting cell division in the shoot apical meristem. Both processes synergistically break dormancy and initiate bud growth [112]. In addition, ERF026 positively regulates JA biosynthesis by directly binding to the promoters of the JA synthesis genes *ALLENE OXIDE CYCLASE* (*AOC*)*, 12-OXO-PHYTODIENOIC ACID REDUCTASE* (*OPR*), *OPC-8:CoA LIGASE 1* (*OPCL1*), and *MYC2-like*, thereby inhibiting the plant height, branching, biomass, and drought resistance of *Medicago sativa* L. [113]. All in all, plant hormones play an important role in regulating shoot branching. Different plant hormones have distinct effects.

## 4. Conclusions and Future Perspectives

**Figure 3:**
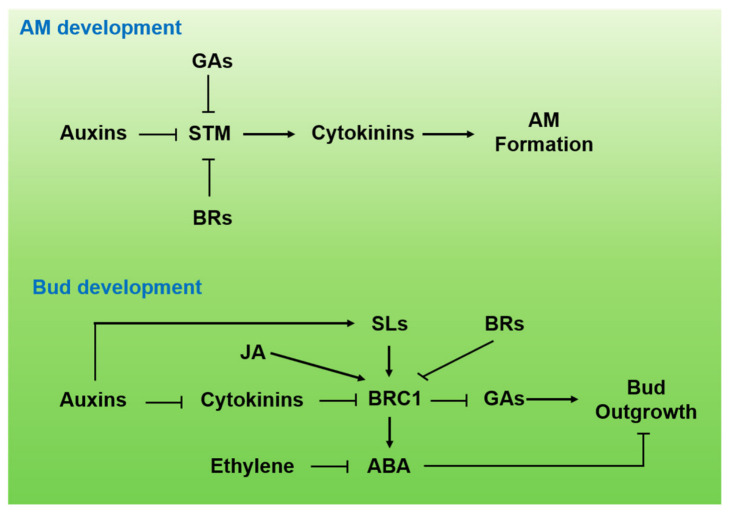
A summary of the effects of different phytohormones on shoot branching. In AM development stage, auxin inhibits the initiation of AM by suppressing the expression of *STM*, which in turn inhibits CK synthesis in AM. Similarly, GAs and BRs exhibit analogous effects to auxin. In bud development stage, BRC1 serves as a crucial inhibitory factor. CKs and BRs promote shoot branching by inhibiting *BRC1* expression, whereas JAs, SLs, and auxin inhibit shoot branching by promoting *BRC1* expression. GAs and ABA act downstream of BRC1, with GAs promoting but ABA inhibiting shoot branching. Furthermore, ethylene can enhance shoot branching by inhibiting ABA synthesis. Lines with arrows and blunt ends denote promotion and repression, respectively.

In conclusion, phytohormones play a crucial role in shoot branching regulation. During the AM development stage, auxin, GAs, and BRs modulate CK synthesis by inhibiting *STM* expression, thereby suppressing AM formation. As the plants transition into the bud development stage, BRC1, a key inhibitor of bud development, restricts bud outgrowth by downregulating GAs and promoting ABA synthesis, while ethylene functions to inhibit ABA’s effects. CK and BRs act upstream of *BRC1* to suppress its expression, thus playing a positive role in shoot branching. Conversely, auxin, SLs, and JAs negatively regulate shoot branching by enhancing *BRC1* expression. Notably, the influence of auxin on bud development may not be direct; rather, it is contingent upon the presence of CKs and SLs (Figure 3).

To date, the regulatory mechanisms of shoot branching have been extensively studied. Multiple key genes involved in AM formation, as well as the formation, activation, and sustained outgrowth of axillary buds, have been identified through the regulation of hormones and their signaling pathways, and these regulatory mechanisms have gradually become clearer. However, in the hormonal regulation of shoot branching, the functions of certain hormones (such as ethylene, JAs, and SA) have not yet been fully elucidated, especially under specific conditions (e.g., insect feeding, virus infection, etc.), and their integration nodes with other hormone signaling pathways remain unclear.

As important participants in the regulation of gene expression, the precise spatiotemporal expression patterns of transcription factors during branching regulation still require further in-depth investigation. Additionally, it is noteworthy that the hormonal regulation of shoot branching has largely focused on the transcriptional level and protein–protein interactions, while research at the post-transcriptional level remains very limited. Non-coding RNAs, as a class of RNA molecules with regulatory functions, play important roles at the post-transcriptional level. Moreover, RNA modifications regulate the entire RNA lifecycle, including processing, splicing, stability, translation, and localization, and are crucial for coordinating gene expression programs during dynamic transitions. Systematically identifying non-coding RNAs (such as miRNAs, circular RNAs, long non-coding RNAs, etc.) and RNA modifications (m^6^A, m^5^C, m^1^A, m^7^G, etc.) using multi-omics platforms during plant branching development, and elucidating the molecular mechanisms by which they finely regulate lateral branch development, is of great significance for further revealing post-transcriptional regulation of plant branching development.

Lateral branches are critical agronomic traits in crops. Therefore, elucidating the molecular mechanisms underlying phytohormone-mediated shoot branching regulation is essential for the precise regulation of related gene expression through gene editing technologies. This approach can facilitate the genetic improvement of crops, enhancing both yield and quality. On the one hand, CRISPR-Cas9-mediated gene editing technology can be employed to knock out the binding sites of specific transcription factors (e.g., BZR1) on the *BRC1* promoter, thereby alleviating their inhibitory effects on BRC1. On the other hand, this technology can also be utilized to knock in cis-elements that are recognized by specific transcription factors (e.g., MYC2) into the *BRC1* promoter to enhance its expression, ultimately achieving the precise genetic enhancement of crop plant architecture.

## Data Availability

All data are included in the article.

## Abbreviations

The following abbreviations are used in the manuscript:

ABA	Abscisic acid
AM	Axillary meristems
BES1	BRI1-EMS-SUPPRESSOR 1
BRC1	BRANCHED 1
BR	Brassinosteroid
CCD7/8	Carotenoid cleavage dioxygenases 7/8
CK	Cytokinin
CUC	CUP-SHAPED COTYLEDON
D14	DWARF 14
D53	DWARF 53
DRN	DORNROSCHEN
GA	Gibberellin acid
JA	Jasmonic acid
LAS	LATERAL SUPRESSOR
MAX2/3/4	MORE AXILLARY GROWTH 2/3/4
MYC2	MYELOCYTOMATOSIS PROTEINS 2
miRNA	MicroRNA
MOC1	MONOCULM 1
PIN3/4	PIN-FORMED 3/4
PAT	Polar auxin transport
RAX	REGULATOR OF AXILLARY MERISTEMS
REV	REVOLUTA
SL	Strigolactone
SMXL	SUPPRESSOR OF MORE AXILLARY GROWTH 2 LIKE
SPL	SQUAMOSA PROMOTER-BINDING-PROTEIN-LIKE
STM	SHOOT MERISTEM-LESS
WUS	WUSCHEL
